# 3D Printing of a Multi-Layered Polypill Containing Six Drugs Using a Novel Stereolithographic Method

**DOI:** 10.3390/pharmaceutics11060274

**Published:** 2019-06-11

**Authors:** Pamela Robles-Martinez, Xiaoyan Xu, Sarah J. Trenfield, Atheer Awad, Alvaro Goyanes, Richard Telford, Abdul W. Basit, Simon Gaisford

**Affiliations:** 1 Department of Pharmaceutics, UCL School of Pharmacy, University College London, 29–39 Brunswick Square, London WC1N 1AX, UK; pamela.martinez.13@ucl.ac.uk (P.R.-M.); xiaoyan.xu.13@ucl.ac.uk (X.X.); sarah.trenfield.16@ucl.ac.uk (S.J.T.); atheer.awad.15@ucl.ac.uk (A.A.); 2 FabRx Ltd., 3 Romney Road, Ashford TN24 0RW, UK; a.goyanes@fabrx.co.uk; 3 Departamento de Farmacología, Farmacia y Tecnología Farmacéutica, R + D Pharma Group (GI-1645), Universidade de Santiago de Compostela, 15782 Santiago de Compostela, Spain; 4 School of Chemistry and Forensic Sciences, University of Bradford, Richmond Road, Bradford BD7 1DP, UK; R.Telford@bradford.ac.uk

**Keywords:** three-dimensional printing, fixed-dose combinations, additive manufacturing, 3D printed drug products, printlets, tablets, personalized medicines, multiple-layer dosage forms, stereolithography, vat polymerisation

## Abstract

Three-dimensional printing (3DP) has demonstrated great potential for multi-material fabrication because of its capability for printing bespoke and spatially separated material conformations. Such a concept could revolutionise the pharmaceutical industry, enabling the production of personalised, multi-layered drug products on demand. Here, we developed a novel stereolithographic (SLA) 3D printing method that, for the first time, can be used to fabricate multi-layer constructs (polypills) with variable drug content and/or shape. Using this technique, six drugs, including paracetamol, caffeine, naproxen, chloramphenicol, prednisolone and aspirin, were printed with different geometries and material compositions. Drug distribution was visualised using Raman microscopy, which showed that whilst separate layers were successfully printed, several of the drugs diffused across the layers depending on their amorphous or crystalline phase. The printed constructs demonstrated excellent physical properties and the different material inclusions enabled distinct drug release profiles of the six actives within dissolution tests. For the first time, this paper demonstrates the feasibility of SLA printing as an innovative platform for multi-drug therapy production, facilitating a new era of personalised polypills.

## 1. Introduction

Multiple drug therapies have gained increasing attention in healthcare because of improved treatment outcomes for diseases with complex pathologies, such as HIV-1 infection, hypertension, tuberculosis and type II diabetes mellitus [1,2,3,4]. Despite this, polypharmacy (involving the administration of five or more medicines) is arguably the most pressing prescribing issue, linked to increasing rates of non-adherence and patient confusion due to the high pill burden and complex administration requirements [5]. Such challenges can be overcome by utilising fixed-dose combinations (FDCs) or polypills, whereby more than one drug is incorporated into the same drug product [6,7,8]. Indeed, the commercially available polypill (Polycap^TM^), which contains enteric-coated aspirin, ramipril, simvastatin, atenolol, and hydrochlorothiazide, has been shown to be effective in reducing multiple cardiovascular risk factors [9,10].

The main barrier to the widespread introduction of FDCs, however, lies in their manufacturing and lack of flexibility in dosing. Conventional powder compaction typically produces homogeneous tablets containing fixed strengths on a large commercial scale, an approach that is wholly unsuitable for therapies that require flexibility in dosing or drug combination(s). For example, if a patient requires a change in dose and/or drug whilst maintained on a FDC, often the treatment will have to be withdrawn and the patient would be initiated on separate dosage forms [11]. Furthermore, narrow therapeutic index drugs or those that require frequent dose titrations are unsuitable for FDC regimens [12,13]. In the era of personalised medicine, it is clear that a novel platform that enables a flexible process for tailored dosing and drug combinations is required [14,15,16,17].

It is in this niche that three-dimensional printing (3DP) offers significant promise as a transformative technology [18,19,20,21,22,23]. Three-dimensional printing is an additive manufacturing technique that fabricates objects from a computer-aided design (CAD) file in a layer-by-layer manner [24,25,26,27,28]. Owing to its flexibility, 3DP allows the combination of multiple materials in a single dosage form with different geometries [29,30,31,32,33]. Since the drugs are physically separated, it is possible to adjust doses and release profiles individually as well as to co-formulate drugs that may potentially interact [12,34,35,36,37,38]. Indeed, previous studies have 3D printed polypills containing paracetamol and caffeine with varying designs (multi-layered and DuoCaplet), enabling specific release profiles to be attained depending on the position of the drug in the caplet, independent of drug solubility [39]. Khaled. et al. fabricated a 3D printed polypill containing five drugs that were released in two different profiles [40]. The same group incorporated three different drugs within a single 3D printed tablet using a semisolid extrusion-based printer, each of which have a distinct release profile depending on their spatial location [41].

The most widely used 3DP technique in pharmaceuticals is fused deposition modelling (FDM), which involves the use of drug-loaded polymer filaments as feedstock that are heated and deposited layer-by-layer [42,43,44,45,46,47]. However, most commercially available FDM printers can only print with a limited number of filaments, and hence, enabling a maximum deposition of a limited number of spatially separated drugs [35]. Stereolithographic (SLA) 3DP is an alternative technology hitherto relatively unexplored for pharmaceutical applications. It works by using a laser to photocure a liquid resin, comprising a photopolymerisable monomer and a photoinitiator that upon exposure to light initiates polymerisation of the monomer [48]. Stereolithographic 3DP offers some key advantages over other 3DP technologies including avoidance of thermal degradation [28,49], improved resolution and higher accuracy [50], and is also considered a faster method than FDM or selective laser sintering (SLS) 3DP processes [51]. Further information about the SLA process has been described elsewhere [52].

By blending a drug into the resin, SLA 3DP has previously been used to make tablets [52] and hydrogels [53] and its versatility has allowed the exploration of how geometric parameters influence drug release kinetics [54]. Thus far, however, no such work has demonstrated the ability for SLA to produce polypills, likely due to the difficulty in printing spatially-separated layers. A particular challenge relates to the software and hardware of commercially available SLA printers, which does not allow for multi-resin printing.

In this work, for the first time, we have overcome this limitation of SLA printing by developing an SLA printer that is capable of printing multi-layered tablets. We exemplify its use by printing a polypill 3D-printed tablet (printlet) containing six different model drugs (paracetamol, naproxen, caffeine, aspirin, prednisolone, and chloramphenicol), some of which are commonly administered together to improve their efficacy. The spatial separation of the drugs was determined with Raman microscopy and the modification of drug release rates upon changing polypill geometry (cylindrical and ring shapes) and excipient addition was evaluated using dissolution tests. Critically, this work has generated a new SLA pharmaceutical printing process, revolutionising the manufacture of polypills and treatment pathways for patients.

## 2. Materials and Methods 

The model drugs paracetamol (MW = 151.2 g/mol), acetylsalicylic acid (MW = 180.2 g/mol), naproxen (MW = 252.2 g/mol), chloramphenicol (MW = 323.1 g/mol), and caffeine (MW = 194.2 g/mol) were purchased from Sigma–Aldrich Ltd. (Gillingham, UK) and prednisolone (MW = 360.4 g/mol) was purchased from Severn Biotech Ltd. (Kidderminster, UK).

Polyethylene glycol diacrylate (PEGda, average MW 575 g/mol) and diphenyl (2,4,6-trimethylbenzoyl) phosphine oxide (TPO) were purchased from Sigma-Aldrich Ltd. (Gillingham, UK). The salts for preparing the buffer dissolution media were purchased from VWR International Ltd., Poole, UK. All materials were used as received.

### 2.1. 3D Printing

PEGda was used as the photopolymerisable monomer and TPO as the photoinitiator (PI). The compositions of the formulations are shown in Table 1.

Each formulation was prepared by dissolving the drug and the PI in liquid PEGda and PEG300 when applicable. The components were added into a beaker under constant stirring until complete dissolution of the powders in the polymer(s). Then each solution was poured into a resin tray for printing.

**Figure 1:**
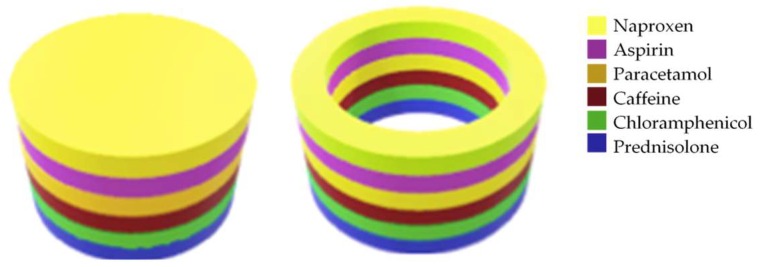
3D designs of the printlets. Type I: Cylinder (**left**, 10 mm diameter and 3 mm height), Types II and III: Ring (**right**, 10 mm diameter and 6 mm height).

All printlets were fabricated using a Form 1+ SLA 3D printer (Formlabs Inc., Somerville, MA, USA). The printer was equipped with a 405 nm laser able to fabricate objects with a resolution of 300 μm and a layer thickness of 25 μm, 50 μm, 100 μm or 200 μm. Printlets (a cylinder—10 mm diameter and 3 mm height, or a ring—10 mm diameter and 6 mm height) were designed in AutoCAD^®®^ 2017 (Autodesk Inc, San Rafael, CA, USA) and exported as a stereolithographic file (.stl) (Figure 1) to the Preform Software v.2.3.3 OpenFL, (Formlabs Inc., Somerville, MA, USA).

The printlets were fabricated keeping the order of the drugs in the polypill unchanged, having the drugs with the higher water solubility (paracetamol and caffeine) in the inner layers, whereas the drugs with the lowest water solubility (naproxen and prednisolone) were printed in the outer layers (Figure 1). Three forms of polypill were printed:

- Type I: Cylinder shape
- Type II: Ring shape
- Type III: Ring shape with a soluble filler (PEG 300)

The Form 1+ printer is designed to print homogeneous objects. To fabricate printlets with different drugs in discrete layers it is necessary to pause printing in order to change the resin formulation in the printing tray. Hence, the use of an application programming interface (OpenFL version of PreForm software) was required to enable the 3D printer to be manually communicated with.

The OpenFL version of the software PreForm was used to allow pausing of printing and raising of the build platform to enable switching of the resin tray. Once the resin tray was changed, the build plate was lowered to its previous position and printing was resumed. The required number of layers (6 blocks of layers of 0.5 mm for the cylinder tablets and 6 blocks of layers of 1 mm for the ring-shaped printlets) was then easily printed, with a deionised water rinse of the printed object between resins to avoid cross-contamination. After this, the platform was returned to its previous position to print the next block of layers until the polypill was completed.

The printlets were printed directly on the build platform at room temperature without supports.

### 2.2. Printlet Dimensions

The printlets were weighed and measured (width and height) using a digital calliper (0.150 mm PRO-MAX, Fowler, mod S 235 PAT). The measurements were performed in triplicate.

### 2.3. Raman Spectroscopy and Mapping

Samples were mounted and focused using a 50× objective on a Renishaw RA802 Pharmaceutical Analyser equipped with a 785 nm laser operating at 50% power (ca. 100 mW at sample). Spectral arrays were acquired with 26,000 spectra recorded over the surface of the sample using a step size of 50 μm in the *x*- (10.15 mm) and *y*- (6.5 mm) dimensions.

Processing was performed with Renishaw WiRE software using two approaches: (i) direct classical least-squares (DCLS) component matching to reference 3D prints of the pure drugs in the printing matrix and (ii) using direct classical least-squares (DCLS) component matching to reference spectra extracted from each of the 6 layers of the printed polypill layers.

Higher spatial resolution maps were acquired across the naproxen, aspirin, and paracetamol layers using the same basic acquisition parameters, with an increased spatial resolution achieved by acquiring spectral arrays with ca. 30,000 spectra recorded over a section of the sample (1 mm along *x* and 3 mm along *y*) using a step size of 10 μm.

### 2.4. X-ray Powder Diffraction (XRPD)

X-ray powder diffraction patterns of pure drugs and individual printed discs (23 × 1 mm) were recorded using a Rigaku MiniFlex 600 (Rigaku, The Woodlands, TX, USA) with a Cu Kα X-ray source (λ = 1.5418 Å) and accompanying software Miniflex Guidance Version 1.2.01. The intensity and voltage applied were 15 mA and 40 kV. The angular range of data acquisition was 3–40° 2θ, with a step size of 0.02° at a speed of 2° min^−1^.

### 2.5. Determination of Drug Concentration in the Polypills

Printlets were crushed using a mortar and pestle with 50 mL of ethanol to enhance extraction of poorly water-soluble drugs, this solution was then taken to 1 L with deionised water and constantly stirred during 24 h. Samples of the solutions were filtered through a 0.45 μm filter (Millipore Ltd., Dublin, Ireland) and the amount of drug in solution was determined using HPLC (Hewlett Packard 1050 Series HPLC system, Agilent Technologies, Cheadle, UK).

The validated HPLC assay consisted of a stationary phase of an Eclipse 5 μm C18 column, 4.6 mm × 150 mm (Agilent, Santa Clara, CA, USA) and a mobile phase with a gradient elution system of ortho-phosphoric acid, pH = 2.7 (A) and acetonitrile (B) at 25 °C. The gradient system consisted of; 0–7.5 min linear change from A–B (87:13 *v*/*v*) to A–B (50:50 *v*/*v*) and kept until 8.5 min; then 8.5–9.5 min linear change to the initial conditions, A–B (87:13 *v*/*v*). The flow rate was kept at 1.5 mL/min and the injection volume was 20 μL. The eluent was screened at a wavelength of 263 nm. The retention times for the drugs were as follows: paracetamol, 2 min; caffeine, 2.6 min; aspirin, 5.2 min; chloramphenicol, 5.8 min; prednisolone, 6.15 min; and naproxen, 9.4 min (so a total elution time of 10 min). All measurements were made in duplicate.

### 2.6. Dynamic Drug Dissolution Testing Conditions

Drug dissolution profiles for the printlets were obtained with a USP II apparatus (Model PTWS, Pharmatest, Germany). The printlets were placed in 750 mL of 0.1 M HCl for 2 h to simulate the gastric compartment, and then transferred into 950 mL of modified Hanks (mHanks) bicarbonate physiological medium for 35 min (pH 5.6 to 7.4); and then in modified Krebs buffer (1000 mL) (pH 7 to 7.4 and then to 6.5). The modified Hanks buffer-based dissolution medium (136.9 mM NaCl, 5.37 mM KCl, 0.812 mM MgSO_4_·7H_2_O, 1.26 mM CaCl_2_, 0.337 mM Na_2_HPO_4_·2H_2_O, 0.441 mM KH_2_PO_4_, 4.17 mM NaHCO_3_) forms an in situ modified Kreb’s buffer by addition of 50 mL of pre-Krebs solution (400.7 mM NaHCO_3_ and 6.9 mM KH_2_PO_4_) to each dissolution vessel [55,56].

The formulations were tested in the small intestinal environment for 3.5 h (pH 5.6 to 7.4), followed by pH 6.5 representing the colonic environment [55,57,58]. The medium is primarily a bicarbonate buffer in which bicarbonate (HCO^3−^) and carbonic acid (H_2_CO_3_) co-exist in equilibrium, along with CO_2_ (aq) resulting from dissociation of the carbonic acid. The pH of the buffer is controlled by an Auto pH System™ [59,60], which consists of a pH probe connected to a source of carbon dioxide gas (pH-reducing gas), as well as to a supply of helium (pH-increasing gas), controlled by a control unit. The control unit is able to provide a dynamically adjustable pH during testing (dynamic conditions) and to maintain a uniform pH value over the otherwise unstable bicarbonate buffer pH.

The paddle speed of the USP-II was fixed at 50 rpm and the tests were conducted at 37 ± 0.5 °C (*n* = 3). Sample of the dissolution media (1 mL) was withdrawn and the drug concentration was determined by HPLC using the method described above.

### 2.7. Determination of Swelling Ratio (SR) for Individual Layers

Three-dimensional printed blocks of layers for each formulation were quickly rinsed with deionised water then blotted with filter paper to remove any uncured liquid formulation and water on the surface immediately following fabrication, then they were weighed (*W_i_*). The cylinders were then placed into 0.1 M HCl for 2 h, then transferred to modified Hanks (mHanks) bicarbonate physiological medium for 22 h at 37 °C to simulate the dissolution test conditions. At specific time points the excess water was carefully wiped off and the layers were weighed (*W_s_*). The SR was calculated using the following equation:

(1) $$SR=\frac{W_{s}}{W_{i}}$$

## 3. Results and Discussion

### 3.1. 3D Printing Process

**Figure 2:**
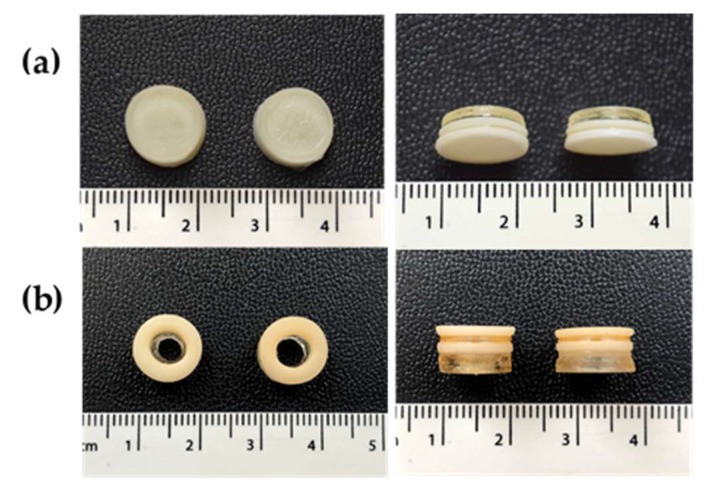
Polypill printlet (**a**) Type I (cylinder shape) and (**b**) Type II (ring shape). The Type III formulation was visually identical to Type II, and hence, has not been included here. The scale is in cm.

For the first time, it was possible to modify a commercial SLA 3D printer in order to fabricate a series of polypill printlets containing six drugs and in unique geometries (Type I: cylindrical and Types II and III: ring-shaped; Figure 2). The commercially available Form 1+ printer has the functionality to only create homogeneous objects composed of single resins, making it impossible for the production of printed dosage forms containing spatially-separated active ingredients. In order to achieve multi-resin printing, it was identified that the printer would need to be paused, the build platform raised, and the resin tray removed and replaced with a new resin formulation. Although the 3D printer PreForm software does have the functionality to pause printing at any point during the fabrication process, the build platform currently remains in the same position (where either the object or the platform itself are within the resin tray), physically obstructing the change of the resin tray or the material within it.

In order to overcome this challenge, we re-designed the printer software to enable a controlled raising and lowering of the build platform once printing was paused, facilitating manual changing of the resin in the printer tray. To achieve this, the Form 1+ printer software was manually modified using the OpenFL version of PreForm software, which is an application programming interface for the Form 1 and Form 1+ FormLabs 3D printers. An application programming interface is a group of functions, commands, protocols, and objects that allows programmers to create software or interact with an external system (the 3D printer in this case) without having to write a code from scratch. Here, the software was modified to include command inputs that enabled the following six steps to be carried out: (1) the resin formulation was printed using SLA; (2) the printing process was paused upon layer completion; (3) the build platform was raised, enabling resin tray removal; (4) the resin tray was replaced which included a different resin formulation; (5) the build plate was lowered to its previous position and; (6) printing was resumed to create the next formulation layer.

In this way, multiple polypill printlets could be easily fabricated in 30 min, with the order of drugs in the layers controlled by the resin formulation in the tank at any particular point. The customised print settings (with six laser passes for the first layer to ensure adhesion and two for the rest) used allowed the successful production of printlets directly on the build platform, achieving good adhesion without significantly affecting the dimensions. Crucially, this approach avoids material wastage and potential dose variation compared with other methods that utilise supports for adhesion to the build platform that need to be removed and discarded post-printing.

### 3.2. Physical Characteristics

#### 3.2.1. Drug Distribution and Solid-State Characteristics

**Figure 3:**
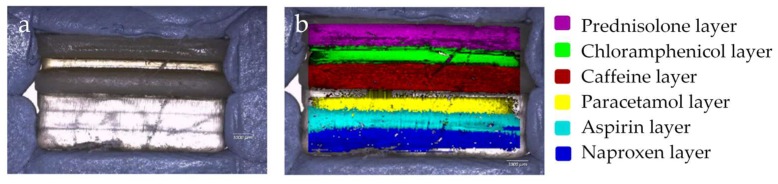
Visual imaging of a Type I polypill, using (**a**) optical light microscopy and (**b**) Raman mapping. The images show the spatial separation of layers.

Raman spectroscopy has previously been used to evaluate the spatial distribution and phase of drugs within tablets, and as such, was used here to map a cross-sectional surface of a multi-layered 3D-printed flat polypill [61]. Processing of the arrays using DCLS component matching to produce false colour representations of distribution shows the presence of the six drugs within the six layers of the polypill (Figure 3), highlighting the success in utilising SLA to print separate resin formulations within separate compartments.

**Figure 4:**
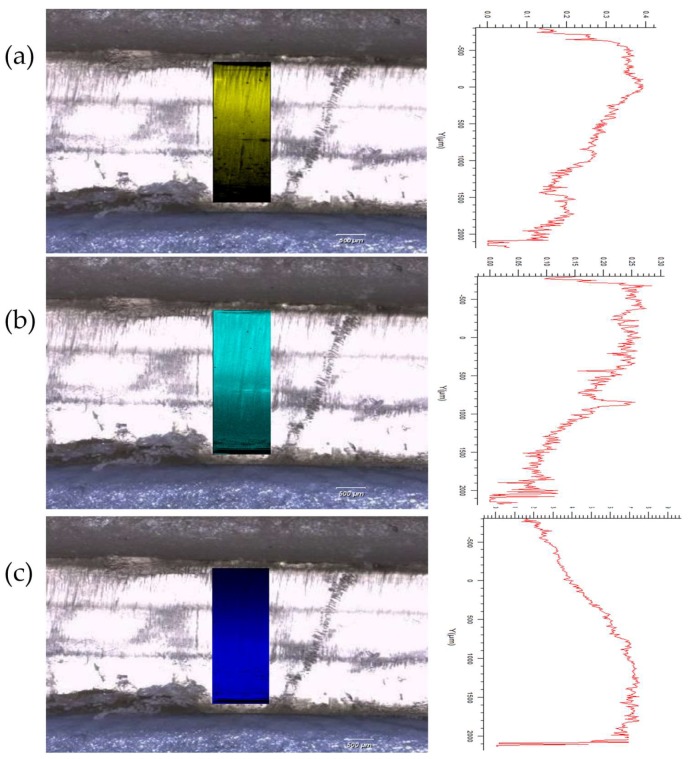
Raman mapping of a Type I polypill across the naproxen, aspirin, and paracetamol layers with an increased spatial resolution (30,000 spectra across 1 mm in *x* by 3 mm in *y*. (**a**) Shows the partial diffusion of paracetamol into the adjacent layers; (**b**) shows the partial diffusion of aspirin into the adjacent layers; and (**c**) shows the partial diffusion of naproxen into the adjacent layers.

However, detailed interrogation of individual Raman spectra within the mapped areas leads us to note that there is evidence of “diffusion” of certain drugs (naproxen, aspirin, and paracetamol) between the layers which was not anticipated through visual examination of the white light microscopic image which shows a distinct boundary between each. Further mapping activities were performed, focusing on the three layers containing naproxen, aspirin, and paracetamol. These spectral arrays were acquired with a significantly higher resolution over a reduced area, i.e., 1 mm in *x* by 3 mm in the *s*-dimensions to better understand the distribution of drug in this area (Section 2.3). Figure 4a–c show DCLS processing of these arrays using pure printed drug references (i.e., containing one drug plus printing matrix) to demonstrate this diffusion effect between these three layers. It is reasonably clear to see that the principle drug content is within the layer containing that drug, but there is evidence of the drug diffusing into the next layers with an attenuating signal, i.e., a diminishing concentration.

**Figure 5:**
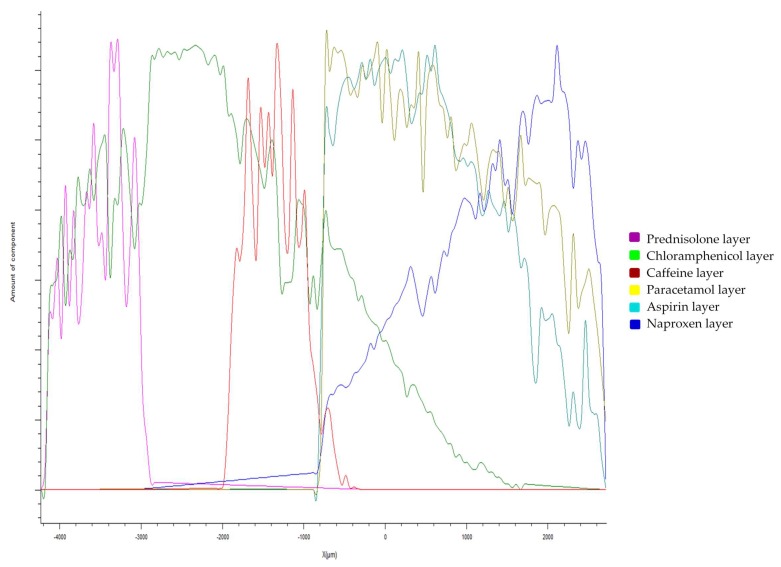
Drug distribution profiles in the *Y*-dimension of the polypill showing the diffusion between layers in the paracetamol, aspirin, and naproxen layers, with a tight distribution in the caffeine and prednisolone layers.

Conversely, caffeine and prednisolone were localised solely within their respective layers, with no evidence of any diffusion. Furthermore, these drugs have appeared to act as a barrier to diffusion of the other layers, e.g., there is no evidence of the paracetamol diffusing into the caffeine layer, whereas it does diffuse into the aspirin layer. Evidence of this differential diffusion effect is presented in Figure 5, where distribution of each drug is evaluated by plotting DCLS match across the *y*-dimension of the mapped polypill.

**Figure 6:**
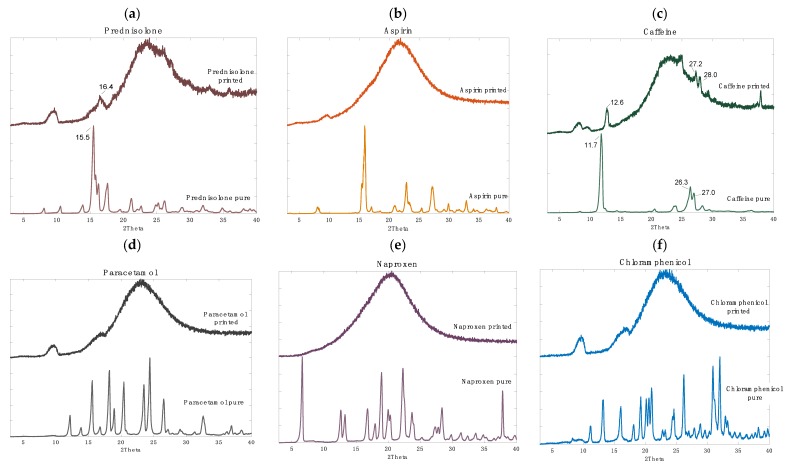
X-ray powder diffractograms of pure and printed drugs: (**a**) prednisolone, (**b**) aspirin, (**c**) caffeine, (**d**) paracetamol, (**e**) naproxen and (**f**) chloramphenicol.

This phenomenon was hypothesised to be due to the phase of the drugs within the printed polypill; post-printing, the layers containing paracetamol, naproxen, aspirin, and chloramphenicol were visually clear with a glassy appearance and the prednisolone and caffeine formulations were white (opaque), which was an initial indicator for differences in solid-state characteristics (Figure 2). These findings were further interrogated using XRPD (Figure 6).

Indeed, four of the drugs (aspirin, paracetamol, naproxen, and chloramphenicol) were found to be in the amorphous phase due to the absence of sharp peaks in the XRPD spectra (Figure 6b,d–f respectively). Conversely, prednisolone and caffeine were found to be present in the crystalline phase (Figure 6a,c respectively). Specifically, several crystalline peaks were found post-printing for caffeine (at 12.6, 27.2, and 28.0 2θ) and for prednisolone, one crystalline peak was observed at 16.4 2θ. In both cases, consistent peak shifts of ~+1 2θ was apparent, which was attributed to the stress–strain influence, or the change in height presentation, of a printed disc versus the raw powder.

Previous studies have highlighted that amorphous drug materials have a higher propensity to diffuse across polymeric matrices [62]. As such, it is likely that in this study the amorphous drugs (aspirin, paracetamol, naproxen, and chloramphenicol) are diffusing across the layers more readily compared with the crystalline drugs (caffeine and prednisolone), which remain in their respective layers. Stabilising drugs in their amorphous phase as a solid dispersion is favourable for low solubility drugs due to the potential for an increase in drug solubility and bioavailability. 

#### 3.2.2. Printlet Dimensions and Weight Variation

In order to evaluate the effect of drug addition on the resin printability, the consistency in weight and dimensions of the polypill printlets was evaluated (target dimensions: 3 mm × 10 mm for the cylinders and 6 mm × 10 mm for the rings) (Table 2). In general, all the formulations yielded slightly wider diameters than their corresponding targets, ranging from 10.73 mm to 11.07 mm. In general, height and weight variation were higher for Type I cylindrical printlets compared with Type II and III ring-shaped printlets. This variability in mass could be due to the multiple factors both from the liquid formulation and the settings of the printer. The number of laser passes for each printed layer and the laser power directly affect the curing depth, and hence, the properties of the printed layer [63]. Hence, the parameters need to be optimised for each resin type. It should also be noted that the difference in the target and real dimensions could be adjusted by simply scaling the electronic object. HPLC was used to evaluate drug content of the polypills post-printing. Drug loading was found to range between 85–104%, which is within the acceptable range for content uniformity (85–115%) set by the British Pharmacopoeia.

### 3.3. Drug Release Studies

**Figure 7:**
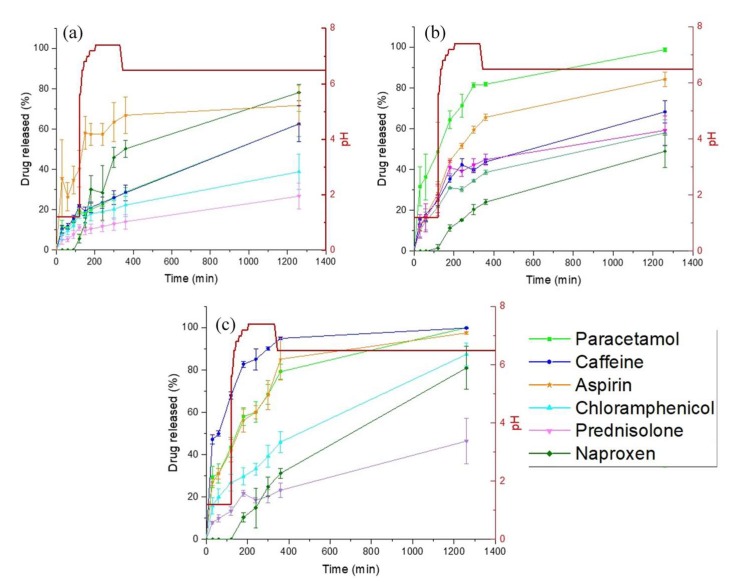
Drug dissolution profiles for the 3D printed polypills: (**a**) Type 1 cylindrical polypill; (**b**) Type II ring-shaped polypill; and (**c**) Type III ring-shaped polypill + PEG 300.

The effect of geometry and excipient addition on drug release was evaluated for each of the three polypill types (Figure 7). For polypill Type I (standard cylindrical shape), none of the drugs reached 100% release after 20 h (range 22–80% release) (Figure 7a). Interestingly, it was observed that the printlets swelled and remained intact at the bottom of the dissolution vessel. SEM images were used to evaluate the surface morphology of the cylindrical polypill before and after swelling (data not shown). Due to the cross-linking of the monomers within the polymeric matrix, the “dry” polypill exhibited a compact surface under SEM imaging, whereas several pores were visible on the surface of a swollen polypill exposed to dissolution medium. These results highlight that the mechanism for drug release from SLA tablets is via diffusion of the water into the printlet, followed by the solubilisation and diffusion of drug through micropore channels within the polymeric matrix.

In order to increase dissolution rate, polypill Type II was designed to have a ring-shaped geometry in order to increase the formulation surface area (and hence dissolution rate). For the water-soluble drugs (placed in the inner layers) the rate of release was enhanced by the presence of a hole in the polypill due to the increased exposure to the dissolution medium (paracetamol 60% versus 95%, and caffeine 60% versus 65% for polypill Type I and II, respectively) (Figure 7b). However, for the lower solubility drugs placed in the outer layers (naproxen and prednisolone), the presence of the hole did not significantly change the drug release profile, likely due to the low water solubility of the drugs.

As such, polypill Type III was formulated and designed in order to evaluate the impact of a soluble filler (PEG 300) on dissolution. PEG 300 is commonly used a solubilising agent in oral formulations, as such, it was hypothesised that its inclusion would increase drug solubility, and hence, release. It was found that the addition of PEG 300 increased drug release substantially compared with polypill Type I (for paracetamol, caffeine, and aspirin ~100% release was achieved after 20 h, and for chloramphenicol and naproxen release was increased to >80%) (Figure 7c). For prednisolone, drug release was increased to 45%; however, it did not achieve complete release after 20 h. It was noted before that poorly soluble drugs like prednisolone are not completely released when incorporated within sustained release devices. This has been attributed mainly to its poor solubility in water and dissolution rate of the drug in a hydrophilic matrix, and, as seen in the XRPD data, could be due to the presence of prednisolone in the crystalline phase (Figure 6) [64].

For prednisolone in particular, Di Colo. et al. noted that the swelling ratio (SR) of the hydrogel had a great influence on the dissolution and diffusion of the drug [65]. To evaluate this concept here, the SR at 24 h and the time to maximum SR was calculated for each of the 3D-printed drug layers (Table 3). The results show that the overall SR values for the different printed layers were similar (~1.1), indicating that the formulations exhibited analogous cross-linking densities despite the difference in drug incorporation. However, the maximum value for swelling ratio for each formulation was reached at different time points (caffeine at 300 min; prednisolone at 240 min; chloramphenicol at 120 min; and the rest at 24 h). Although the maximum SR for the prednisolone layer was reached at 240 min, drug release was not increased beyond this time in the dissolution studies, and hence, in this case was not deemed to be the most significant factor to hindering prednisolone release.

Another possible explanation for the unexpected release behaviour of prednisolone is that it could have a higher affinity for the polymer than for the aqueous media used in the dissolution test. To evaluate this concept, drug diffusion studies were carried out, whereby placebo 3D printed tables were placed into saturated solutions of either prednisolone or caffeine for drug-loading via passive diffusion over a period of 14 days. Results showed that the amount of drug released in fresh media from the loaded 3D-printed tablets was ~3 times higher for prednisolone than for caffeine (data not shown). This suggests that a higher amount of prednisolone was able to diffuse and reside within the cross-linked PEGda networks compared with caffeine, likely due to the higher affinity of prednisolone to the polymer. As well as its poor aqueous solubility, this explains why prednisolone is not readily released from the 3D-printed tablet matrix. Such results highlight the need for continued drug screening and resin formulation optimisation to ensure drug release is appropriately controlled for tailoring to individual patient needs.

## 4. Conclusions

For the first time, a commercial SLA 3D printer was successfully modified to enable multi-resin printing for the fabrication of bespoke and tailored polypills containing six different active ingredients. The printer platform was optimised such that printing could be paused, the resin tray removed, and replaced with different resin formulations. Using this technique, a number of different polypill geometries (cylindrical and ring-shaped) and formulation compositions (with and without a soluble filler) were produced that demonstrated acceptable physicochemical characteristics and differing drug release profiles. Although the drug formulations were suitable for 3D printing, further research is needed to determine the most optimal printer settings for each formulation to achieve accurate dimensions, and thus dosing, and for achieving specific drug release profiles when required. Crucially, this study shows the potential of SLA 3D printing for fabricating multi-layered polypills to improve personalisation for patients.

## Figures and Tables

**Table 1:** Compositions (% *w*/*w*) of the initial resins for printing.

	Formulation	Type I (% *w*/*w*)	Type II (% *w*/*w*)	Type III (% *w*/*w*)
Material				
PEGda		89	89	44.5
PEG300		-	-	44.5
TPO		1	1	1
Drug		10	10	10

**Table 2:** Dimension and weight data for the polypills.

**Type I**		
Width (mm) ± %CV	Height ± SD (mm)	Weight ± SD (mg)
10.99 ± 1.0	2.81 ± 9.8	329 ± 13.6
**Type II**		
Width (mm) ± %CV	Height ± SD (mm)	Weight ± SD (mg)
11.07 ± 0.1	6.12 ± 0.03	501.13 ± 6.3
**Type III**		
Width (mm) ± %CV	Height ± SD (mm)	Weight ± SD (mg)
10.73 ± 0.18	6.12 ± 0.02	553 ± 8.9

**Table 3:** Swelling ratios of the individual 3D-printed layers after 24 h in water. * Indicates maximum swelling occurred before 24 h (caffeine 300 min, prednisolone 240 min, chloramphenicol 120 min).

Drug	Swelling Ratio
Paracetamol	1.21 ± 0.07
Aspirin	1.15 ± 0.03
Naproxen	1.18 ± 0.03
Prednisolone *	1.11 ± 0.05
Chloramphenicol *	1.05 ± 0.02
Caffeine *	1.17 ± 0.02
